# Genotypic variability based association identifies novel non-additive loci *DHCR7* and *IRF4* in sero-negative rheumatoid arthritis

**DOI:** 10.1038/s41598-017-05447-1

**Published:** 2017-07-13

**Authors:** Wen-Hua Wei, Sebastien Viatte, Tony R. Merriman, Anne Barton, Jane Worthington

**Affiliations:** 1 Arthritis Research UK Centre for Genetics and Genomics, School of Biological Sciences, Faculty of Biology, Medicine and Health, Manchester Academic Health Science Centre, University of Manchester, Oxford Road, Manchester, M13 9PT UK; 2 Department of Women’s and Children’s Health, Dunedin School of Medicine, University of Otago, Dunedin, 9016 New Zealand; 3 Department of Biochemistry, University of Otago, PO Box 56 Dunedin, New Zealand; 4 NIHR Manchester Musculoskeletal Biomedical Research Unit, Central Manchester NHS Foundation Trust, Manchester Academic Health Science Centre, Manchester, UK

## Abstract

Sero-negative rheumatoid arthritis (RA) is a highly heterogeneous disorder with only a few additive loci identified to date. We report a genotypic variability-based genome-wide association study (vGWAS) of six cohorts of sero-negative RA recruited in Europe and the US that were genotyped with the Immunochip. A two-stage approach was used: (1) a mixed model to partition dichotomous phenotypes into an additive component and non-additive residuals on the liability scale and (2) the Levene’s test to assess equality of the residual variances across genotype groups. The vGWAS identified rs2852853 (P = 1.3e-08, *DHCR7*) and rs62389423 (P = 1.8e-05, near *IRF4*) in addition to two previously identified loci (*HLA-DQB1* and *ANKRD55*), which were all statistically validated using cross validation. *DHCR7* encodes an enzyme important in cutaneous synthesis of vitamin D and *DHCR7* mutations are believed to be important for early humans to adapt to Northern Europe where residents have reduced ultraviolet-B exposure and tend to have light skin color. *IRF4* is a key locus responsible for skin color, with a vitamin D receptor-binding interval. These vGWAS results together suggest that vitamin D deficiency is potentially causal of sero-negative RA and provide new insights into the pathogenesis of the disorder.

## Introduction

Sero-negative rheumatoid arthritis (RA) is a complex and heterogeneous disorder where patients have no antibodies detected against citrullinated peptides^1^. Sero-negative RA can be considered as a different disorder from sero-positive RA because of a distinct genetic background, e.g. a consistently weaker *HLA-DRB1* association. The (narrow-sense) heritability of sero-negative RA (20%) is much lower than that in sero-positive RA (50%)^2^, suggesting a greater environmental component to susceptibility. Genome-wide association studies (GWASs) of sero-negative RA so far have identified only two loci reaching genome-wide significance (P < 5.0e-08): *HLA-DRB1* and *ANKRD55* ^3, 4^, each conferring relatively moderate effects^5^. It is, therefore, of interest to test for any potential non-additive effects such as gene–environment (GxE) and gene–gene (GxG) interactions that may contribute to the disease heterogeneity.

Thus far efforts in dissecting non-additive signals have yielded only a few convincing examples in human complex phenotypes, each carrying only moderate interaction effects, indicating that any uncovered interactions probably carry moderate/weak effects^6–8^. One obvious reason for the limited findings is that testing for non-additive interactions is more complicated than testing for additive signals, e.g. requiring markers to tag the causal variant in GxG at both loci^6^, and thus requires much larger sample sizes than individual GWAS data offer. Ideally, datasets should also include measurements of environmental exposures of interest, but such data are not always available. Also the genetic markers and/or environmental variables under consideration do not necessarily represent the causal genetic variants and/or environmental factors leading to a reduced power of detection^6^. While causal genetic variants can be captured by deep sequencing data, causal environmental factors are particularly challenging because they are unknown in advance and thus unlikely readily available for GxE interaction tests^9^. Therefore, innovative approaches are needed.

The genotypic variability based genome-wide association study (vGWAS) is a promising approach that can prioritize potentially interacting loci without requiring prior knowledge of interacting factors^10, 11^. vGWAS can achieve this by considering substantial genotypic variability, i.e. differences of phenotypic variation across three SNP genotypes, as the potential aggregated interaction signatures tend to be weak when only additive effects are important but strong when non-additive effects such as GxE interactions are important^12, 13^. Using unused genotypic variability information available in existing GWAS data, vGWAS could provide data complementary to GWAS. It is important to acknowledge, however, that vGWAS loci may not necessarily mark non-additive interaction because other factors such as overdominance and scaling (i.e. various transformations) could also generate apparent genotypic variability^14, 15^. Additional explicit tests of GxE and/or GxG interactions would be needed but only for the identified vGWAS loci, leading to a power increase attributed to a much reduced multiple testing burden^16, 17^. The genotypic association with phenotypic variability approach has been successfully applied to several quantitative traits and has identified interacting loci also carrying major additive effects (i.e. identified in previous GWASs)^16–21^.

We recently adapted vGWAS to analyze dichotomous disease phenotypes using a two-stage approach: firstly a mixed model to partition the dichotomous phenotypes into an additive component and non-additive environmental residuals on the liability scale and then the Levene’s test to assess equality of the residual variances across genotype groups^22^. The vGWAS of sero-positive RA identified the major histocompatibility complex (MHC) as the key interacting locus in addition to the strongest additive signals in three sero-positive RA cohorts^22^. These results collectively indicate that vGWAS can be effective in providing additional insights into the major genetic loci (e.g. MHC) that not only act additively but also interact with other genes and/or environmental factors^23, 24^. The vGWAS observations are also in line with recent GxE simulation studies showing that GxE interactions could be detected even when the environmental exposure was misclassified, if the polygenic risk (i.e. the aggregated additive effects) was used as the G variable^25, 26^.

Nonetheless, vGWAS is yet to reach the potential of identifying loci carrying mainly non-additive effects and ‘novel’ to GWAS. One obvious reason is that vGWAS has not been widely explored in meta-analyses of multiple cohorts. Another possible reason is that such novel loci might be detected in phenotypes with relatively low heritability that are driven mainly by major environmental factors. We therefore performed a vGWAS meta-analysis in sero-negative RA using six independent cohorts from the Rheumatoid Arthritis Consortium International for Immunochip, each genotyped with the Immunochip that is an Illumina Infinium genotyping chip containing 195,806 SNPs for 186 loci known to be involved in any of 12 autoimmune diseases^27^. The cohort samples were recruited in the United Kingdom (UK), Swedish Epidemiological Investigation of Rheumatoid Arthritis (SEE), Swedish Umea (SEU), Netherland (NL), Spain (ES), and United States of America (US), respectively.

## Results

**Figure 1:**
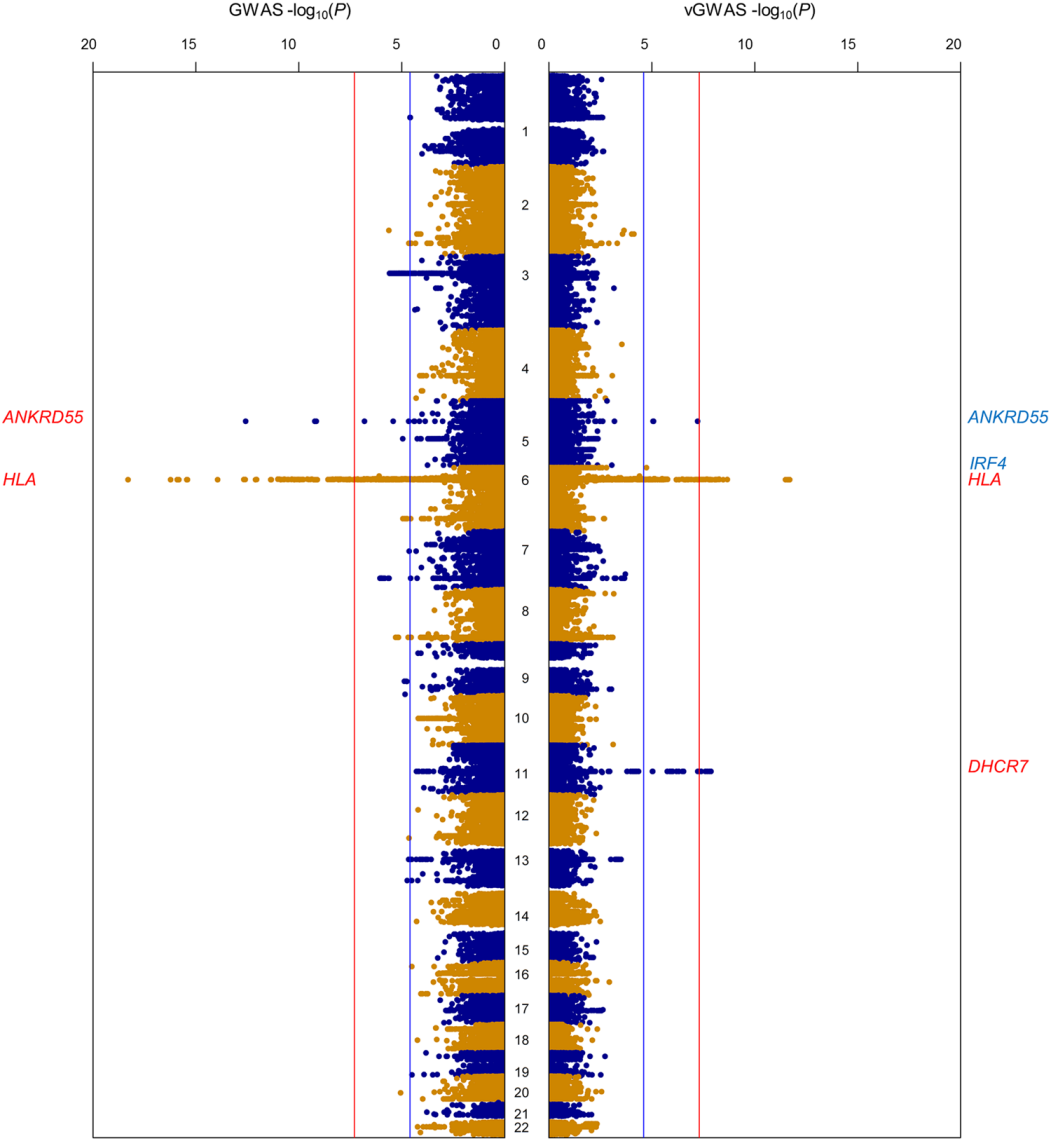
Manhattan plots of GWAS (left) and vGWAS (right) of the combined data in alignment. Each plot uses the -log_10_ scale for P values, red and blue lines for genome-wide and Immunochip-wide significance thresholds respectively, and a panel of gene annotations for loci exceeding the Immunochip-wide threshold in blue and the genome-wide threshold in red.

In total 19,108 unrelated samples (3323 cases and 15,785 controls) and 107,144 autosomal SNPs were included in the vGWAS meta-analysis of the combined data of the six sero-negative RA cohorts (Table 1). The estimate of polygenic heritability was 0.045 suggesting that Immunochip captured only a modest proportion of the total additive variance. The vGWAS identified lead SNPs rs9275428 (P = 2.0e-12, intergenic between *HLA-DQB1* and *HLA-DQA2*) and rs2852853 (P = 1.3e-08, *DHCR7*) at the genome-wide significance level of 5.0e-08, and rs71624119 (P = 5.9e-08, *ANKRD55*) and rs62389423 (P = 1.8e-05, intergenic near *IRF4*) at the Immunochip-wide level of 2.5e-05 (Table 2). These vGWAS signals all carried additive signals as well but only the lead SNPs of *HLA-DQB1* and *ANKRD55* were genome-wide significant in the conventional GWAS where the effects of gender and cohort were corrected (Table 2, Fig. 1). The vGWAS quantile-quantile (QQ) plot suggested no inflation (Supplementary Figure S1). No genome-wide significant signals were detected in the vGWASs of each individual cohort.

**Table 1:** Summary information of study cohorts and the combined data.

cohort	case	control	male%	location
UK	997	8414	55.0	Europe
US	593	2118	67.4	America
SEE	982	1927	72.1	Europe
SEU	237	941	68.7	Europe
NL	299	1991	45.3	Europe
ES	215	394	68.0	Europe
combined	3323	15785	59.5	mixed

**Table 2:** vGWAS signals identified in the combined data and their corresponding GWAS signals.

SNP	chromosome	position	Gene	vGWAS	GWAS
rs71624119	5	56 144 903	*ANKRD55*	5.9e-08	2.6e-13
rs62389423	6	421 281	near *IRF4*	1.8e-05	7.3e-03
rs9275428	6	32 703 201	near *HLA-DQB1*	2.0e-12	1.5e-24
rs2852853	11	71 439 171	*DHCR7*	1.3e-08	5.1e-05

**Figure 2:**
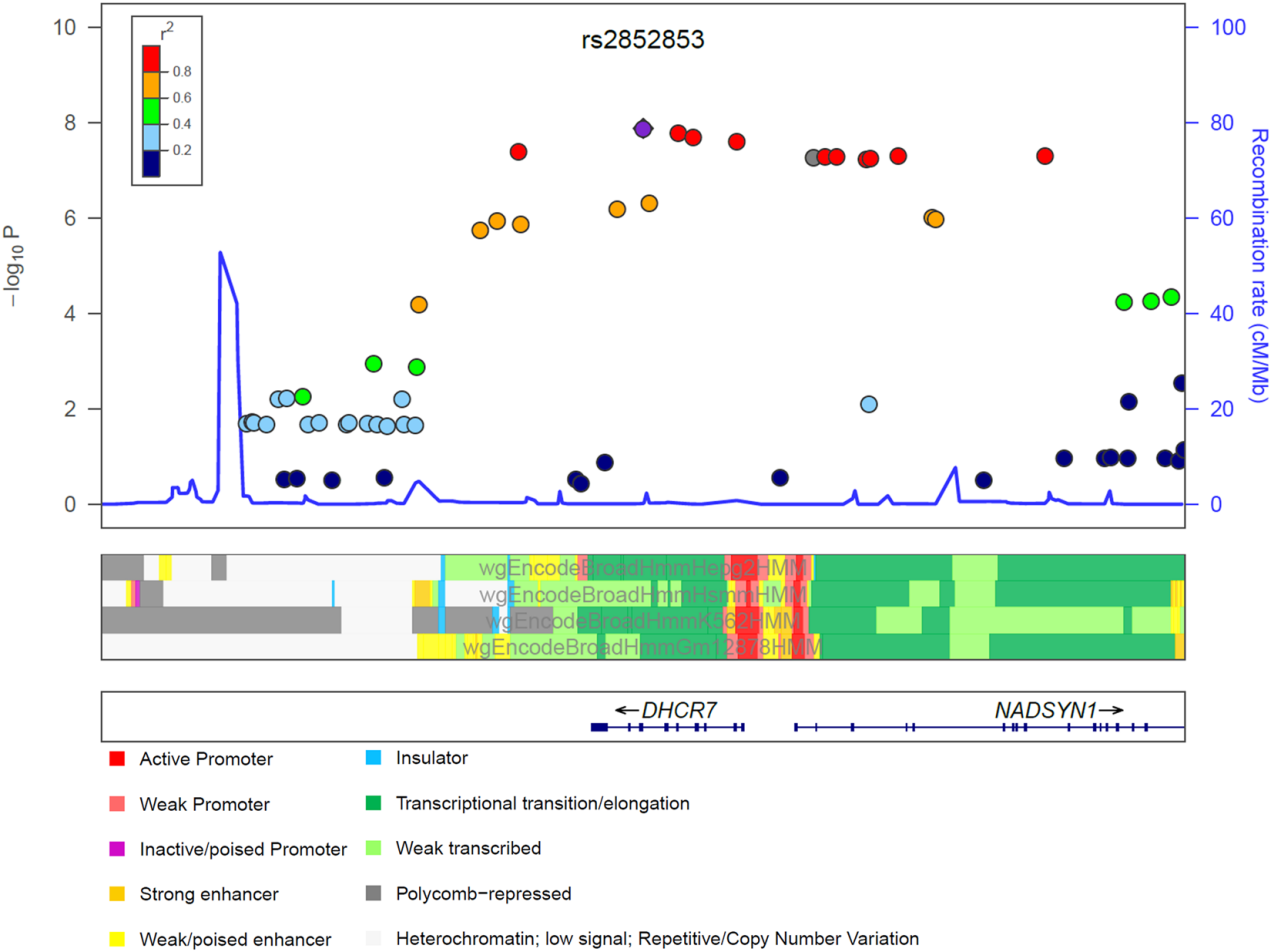
A regional view of *DHCR7*-integrated vGWAS results with ENCODE regulatory annotations.

We used cross validation for statistical validation of the four vGWAS lead SNPs in ten iterations, where the combined data were randomly split into two halves to be used as either discovery or replication per iteration. The *HLA-DQB1* and *ANKRD55* SNPs were discovered ten times at the genome-wide level; the *DHCR7* SNP was discovered nine times at the genome-wide level (ten times at the Immunochip level) whereas the *IRF4* SNP was discovered only two times at the Immunochip-wide level; all the four loci had evidence of statistical validation (P < 0.05) in every iteration (Supplementary Table S1). Of the four vGWAS signals only the *DHCR7* lead SNP rs2852853 was within a conserved region, with a normalized score assigned by UCSC Genome Browser of 542 calculated using the regional annotation in ANNOVAR^28^ and 46-way annotation track. This is in line with the observation of low recombination within the entire *DHCR7* gene, where the majority of the vGWAS important SNPs were highly correlated with rs2852853, mapping to mostly transcriptional elements and also some regulatory elements such as enhancers or promoters (Fig. 2).

We explicitly tested GxG and GxE interactions using each of the identified vGWAS signals as the G and each available environmental factor as the E in the combined data. We found the *DHCR7* lead SNP rs2852853 interacted with cohort (P = 2.0e-02) as well as gender (P = 2.6e-03), and the *HLA-DQB1* lead SNP rs9275428 interacted with cohort (P = 6.1e-04), where cohort and gender were both highly significant environmental factors in the logistic regression models based on Equation 2 in the Methods section (P < 1.0e-183 and P < 1.0e-64 respectively). Indeed, the distributions of the residuals were substantially different across the six member cohorts (Supplementary Figure S2) and between cases and controls (Supplementary Figure S3). When adjusted for multiple tests of four vGWAS signals per E (P = 0.05/4), these GxE interactions were either insignificant or marginally significant each carrying only weak/moderate effects in general. We found no statistically significant GxG interactions.

## Discussion

We show for the first time that vGWAS can identify novel non-additive loci in complex diseases. It is also the first time that vGWAS has proven genetically more informative than GWAS in a disease phenotype with low heritability. This was possible because of meta-analysis of multiple cohorts where cohorts represented geographical locations that might capture certain causal influence on sero-negative RA. The *DHCR7* signals were statistically validated and were involved in interactions with cohort and gender. In addition, the vGWAS identified *HLA-DQB1*, a well-known GWAS locus involved in GxE interactions with cohort and epistatic local interactions within the MHC as previously reported^22^. Such GxE and GxG interactions were not potent and thus unlikely to be identified from conventional locus-by-locus genome-wide scans for non-additive interactions because of the multiple testing burden. Our results therefore support vGWAS as an innovative tool to detect non-additive loci from widely available GWAS data.

*DHCR7* is a genome-wide significant locus associated with vitamin D deficiency and circulating vitamin D levels^29, 30^. *DHCR7* encodes an enzyme 7-dehydrocholesterol reductase that plays an important role in cutaneous synthesis of vitamin D^29–31^. *DHCR7* mutations are believed to be one of the key factors allowing early humans to adapt to Northern latitudes (e.g. Sweden, with inadequate sunlight) by generating additional cutaneous vitamin D3^31^. Although Vitamin D deficiency is a common problem in RA patients, *DHCR7* is moderately associated with sero-positive RA in the UK (i.e. rs4944076, P = 0.008, odds ratio 1.14) when considering only additive effects^32^. Here we also found only a moderate association of *DHCR7* additive effects with sero-negative RA. These GWAS results, together with our vGWAS results, clearly support the hypothesis that aspects of vitamin D metabolism are causal of RA.

Latitude-dependent changes in cutaneous vitamin D3 levels may also be driven by pigment-based mechanisms (e.g. the lightest skin color and lack of ultraviolet-B exposure in northern Europe) and/or plausible mutations in other genes^33, 34^. Of relevance, vGWAS of the combined data identified not only *DHCR7* but also *IRF4* which is a key locus responsible for skin color^35, 36^ with a vitamin D receptor binding interval^37^. These vGWAS results together revealed potentially important biological insights into pathogenesis and heterogeneity of sero-negative RA at least in northern Europe.

This study also raised questions. First, are there any hidden causal environmental factors jointly driving the vGWAS signals in sero-negative RA? Because the additive genetic effects and the effects of gender and cohort were removed at the first stage, the observed vGWAS signals could come only from unsolicited but associated environmental effects and non-additive effects. Given that only limited GxE interactions with cohort and gender were identified, our data seem to support existence of such causal factors and/or their GxE interactions with the vGWAS loci such as *DHCR7*. Dissection of such hidden factors would require further genetic and ecological epidemiological studies but may greatly improve understanding of the etiology of sero-negative RA. Second, is it time to consider modeling macro environmental factors (e.g. distinct geographic locations) in GxE interaction studies? Such macro environmental factors are likely causal of human adaptation and hence induce GxE interactions that can hardly be detected when considering only one environment but can become obvious when analyzing data from various environments together. Further studies will be required to address the questions above. In conclusion, we have demonstrated the potential of vGWAS analysis to identify novel loci for complex diseases.

## Materials and Methods

### Study cohorts, genotyping and quality control

The six sero-negative RA cohorts used in this study have been described in detail elsewhere^3^. Briefly, the cohorts were recruited in four European countries: the United Kingdom, Sweden, Netherland, Spain, and the United States of American respectively. All RA patients fulfilled the 1987 criteria of the American College of Rheumatology and were negative for anti-citrullinated peptide antibody tests. All participants provided written informed consent for participation. This study was approved by the North West Research Ethics Committee (MREC 99/8/84). All experiments (e.g. genotyping) were performed in accordance with relevant guidelines and regulations.

SNPs on the sex chromosomes and samples of non-European origin were excluded. Quality control was conducted for each cohort to ensure: minor allele frequency > 0.01, SNP call rate > 0.95, sample call rate > 0.99, deviation from Hardy–Weinberg Equilibrium P < 1.0e-04. The six cohorts were merged into a combined data based on the common set of SNPs.

### Two-stage vGWAS for case-control disease phenotypes

The two-stage approach previously developed for case-control disease phenotypes^22^ was used to conduct vGWAS analyses for each individual cohort and the combined data. Stage one: a mixed model implemented in GCTA^38^ was used to compute the genetic relationship matrix (GRM) and subsequently the first ten principal components (PCs), and then to predict polygenic liability risk for each unrelated individual by imposing a GRM relatedness threshold of 0.15 recommended for the Immunochip^39^ while setting the disease prevalence as 0.01 and fitting gender and the first 10 PCs (and cohort in the combined data) as covariates in the mixed model. The parameters used in the GCTA command were: *gcta* *--reml --grm-gz --pheno --mpheno --reml-pred-rand --grm-cutoff --prevalence --covar --thread-num --out*.

Stage two: the resultant residuals were tested for variance heterogeneity across three SNP genotypes using the Levene’s (Brown-Forsythe) test implemented in an R package VariABEL^40^:

1 $${T}^{2}=\frac{(N-k){\sum }_{j=1}^{k}\,{n}_{j}({Z}_{j}.-Z\mathrm{..}{)}^{2}}{(k-1){\sum }_{i=1}^{N}\,({Z}_{i}-{Z}_{gi}.{)}^{2}}$$

where the residuals were the trait ***y***; ***Z***_***i***_ = |***y***_***i***_ − ***ỹ***_***gi***_| is the deviation of ***y*** of the ***i*** ^***th***^ sample (***y***_***i***_) and the median of ***y*** in samples with genotype ***g*** (***ỹ***_***gi***_); ***N*** is the sample size and ***k*** is the total possible genotypes; ***n***_***j***_ is the number of samples with genotype ***j***; ***Z***_*j*_. is the mean deviation from the median for genotype ***j*** and ***Z***.. is the mean deviation from the overall median. When ***N*** is large, *T* ^*2*^ is an approximate χ^2^ test taking two degrees of freedom.

The Levene’s (Brown-Forsythe) test requires no assumption of normally distributed phenotypes and hence is suitable for vGWAS of case-control disease phenotypes. For simplicity, the GWAS consensus threshold of 5.0e-08 is adopted as the genome-wide significance threshold, and a threshold of 2.5e-05 previously derived from permutation^22^ is used as the Immunochip-wide suggestive threshold.

### Cross validation

Given a vGWAS signal *G* from a cohort with *N* cases and *M* controls, the following steps were used to cross validate *G*:

- to randomly select 50% *N* and 50% *M* and split the cohort into two independent set1 and set2
- to perform vGWAS for both sets
- to record the P values of the vGWAS signal *G* and check whether it was discovered at either the genome-wide or Immunochip level and/or statistically validated (i.e. the same SNP with a vGWAS P < 5.0e-02)
- to repeat the above (a to c) steps 10 times and count successes of discovery and replication

### Interaction tests

We used the following logistic regression model to test GxE or GxG interactions for an identified vGWAS SNP *G*:

2 $$\mathrm{log}(\frac{p}{1-p})=\mu +\beta X+{\beta }_{g}G+{\beta }_{f}F+{\beta }_{gf}GF+e$$

where *p* is the probability of an individual being a case rather than a control in a population, *µ* is the model constant, *β* is the effects of fixed covariates (e.g. gender and/or cohort), *β*_*g*_ is the effect of a vGWAS SNP *G*, *β*_*f*_ is the effect of the interacting factor *F* that can be an environmental factor (i.e. testing GxE) or another SNP (i.e. testing GxG), *β*_*gf*_ is effect of interactions between *G* and *F*, *e* is the random error.

The *β*_*gf*_ = 0 hypothesis test took 2 degrees of freedom (df) for interactions between *G* and gender, 10 df between *G* and cohort in the combined data and 8 df in the reduced data, 4 df for GxG between *G* and another Immunochip SNP.

### Variant annotation and visualization

ANNOVAR^28^ was used to annotate the identified vGWAS SNPs and to calculate their conservation scores. Enlight^41^ was used to generate plots of vGWAS regions (50 kilobases flanking the lead SNP) of interest.

### Data Availability

The datasets generated during and/or analysed during the current study are available from the corresponding author on reasonable request.

## Electronic supplementary material

Supplementary Figures and Table
