# Supplementary material for: Genotypic variability based association identifies novel non-additive loci DHCR7 and IRF4 in sero-negative rheumatoid arthritis
Source: Sci Rep. 2017 Jul 13;7:5261. doi: 10.1038/s41598-017-05447-1 (PMC5509675; doi:10.1038/s41598-017-05447-1)
Supplement: Supplementary file 1 — Supplementary Figures and Table [file 41598_2017_5447_MOESM1_ESM.doc]

Genotypic variability based association identifies novel non-additive loci *DHCR7* and *IRF4* in sero-negative rheumatoid arthritis

Wen-Hua Wei,^1,2,*^ Sebastien Viatte,^1^ Tony R Merriman,^3^ Anne Barton,^1,4^ Jane Worthington^1,4^

^1^Arthritis Research UK Centre for Genetics and Genomics, School of Biological Sciences, Faculty of Biology, Medicine and Health, Manchester Academic Health Science Centre, University of Manchester, Oxford Road, Manchester M13 9PT, UK

^2^Department of Women’s and Children’s Health, Dunedin School of Medicine, University of Otago, Dunedin 9016, New Zealand

^3^Department of Biochemistry, University of Otago, PO Box 56, Dunedin, New Zealand

^4^NIHR Manchester Musculoskeletal Biomedical Research Unit, Central Manchester NHS Foundation Trust, Manchester Academic Health Science Centre, Manchester, UK

*Correspondence:

WHW ([wenhua.wei@otago.ac.nz](mailto:wenhua.wei@otago.ac.nz))

**
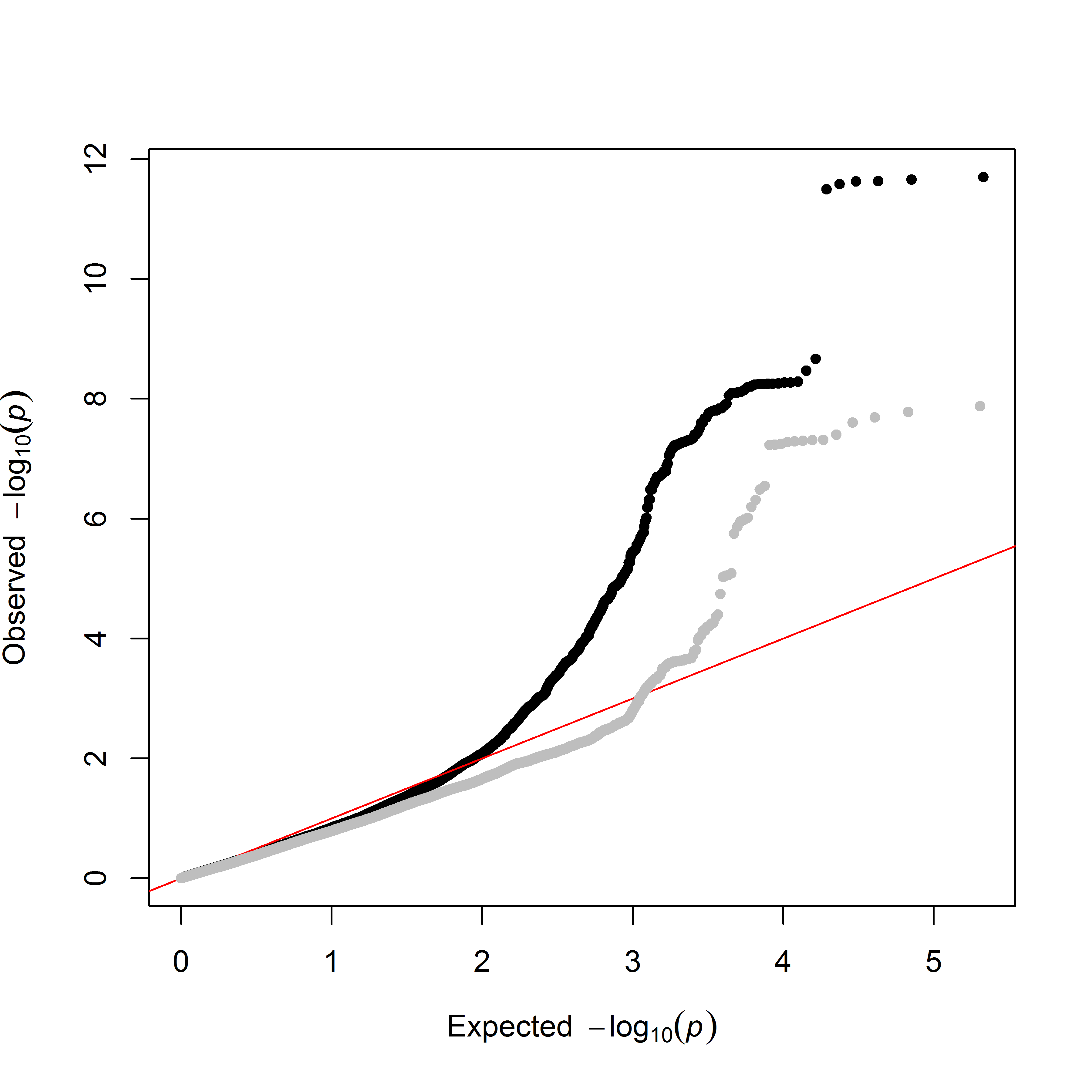
**

Supplementary Figure S1: Quantile-quantile plot of the vGWAS analysis of the combined data (all data in black, exclusion of MHC in grey).


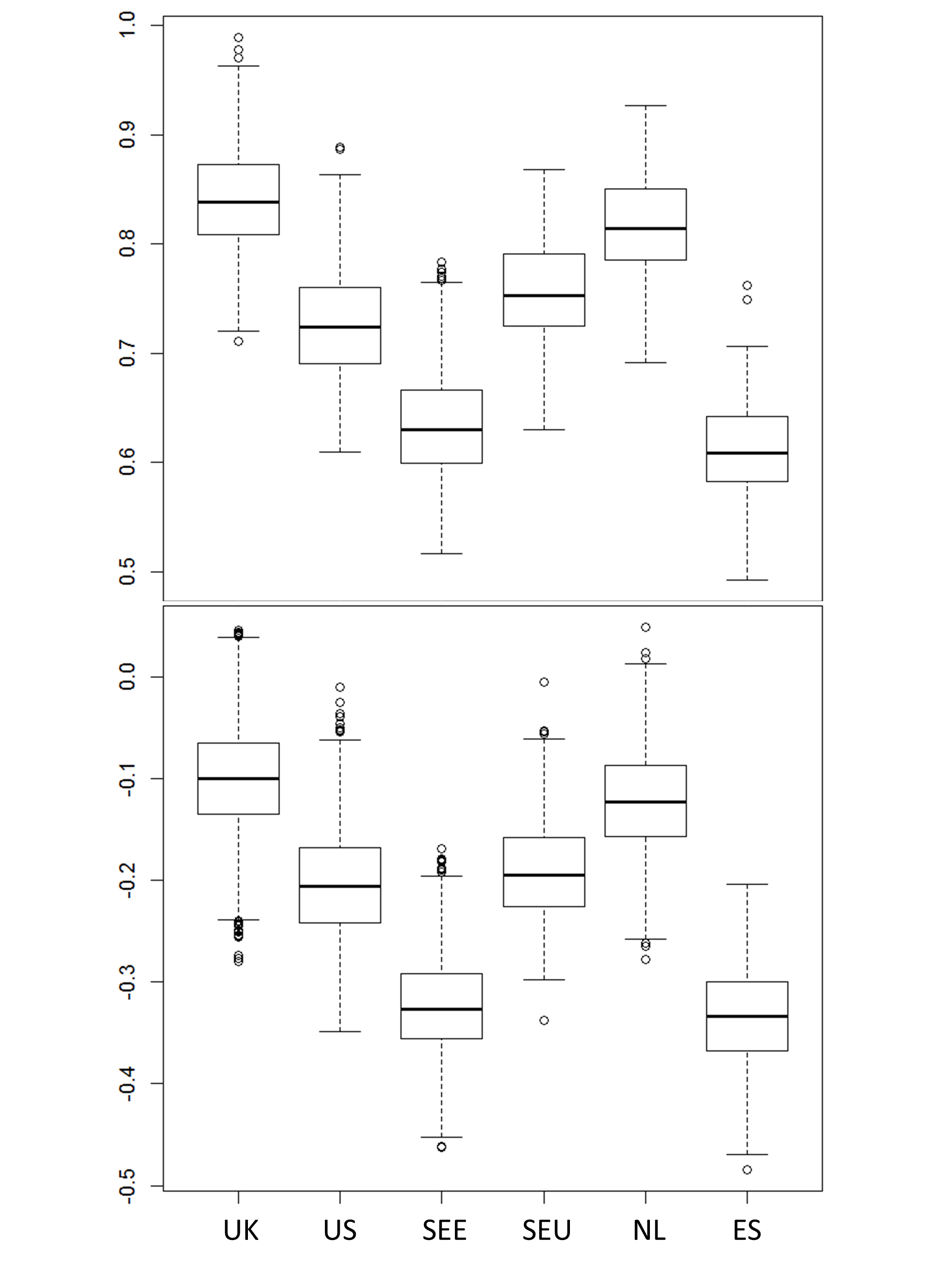


Supplementary Figure S2: Box plots of residuals on the liability scale in cases (up) and controls (down) of six member cohorts. UK: the United Kingdom; SEE: Swedish Epidemiological Investigation of Rheumatoid Arthritis; SEU: Swedish Umea; NL: Netherland; ES: Spain; US: the United States of America.


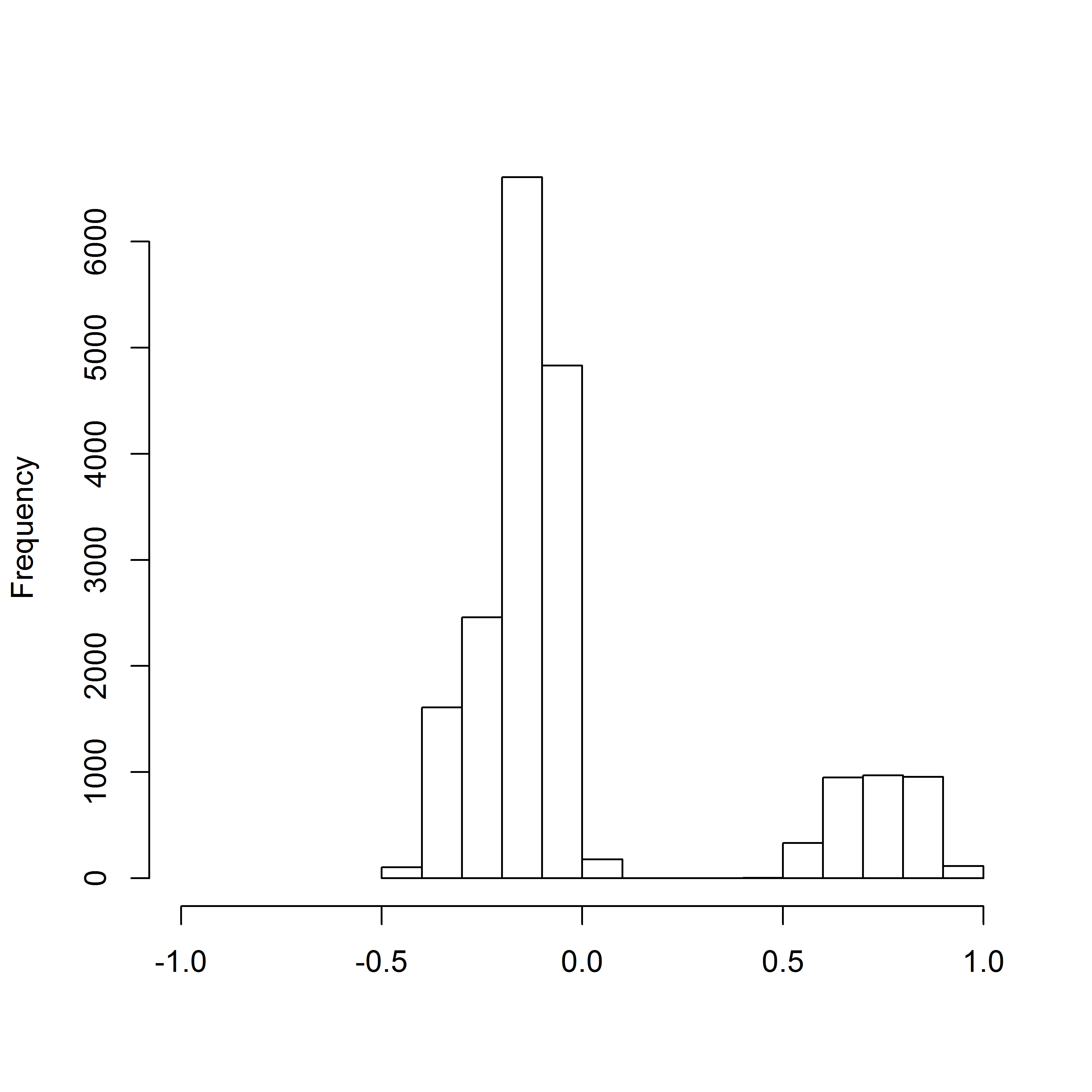

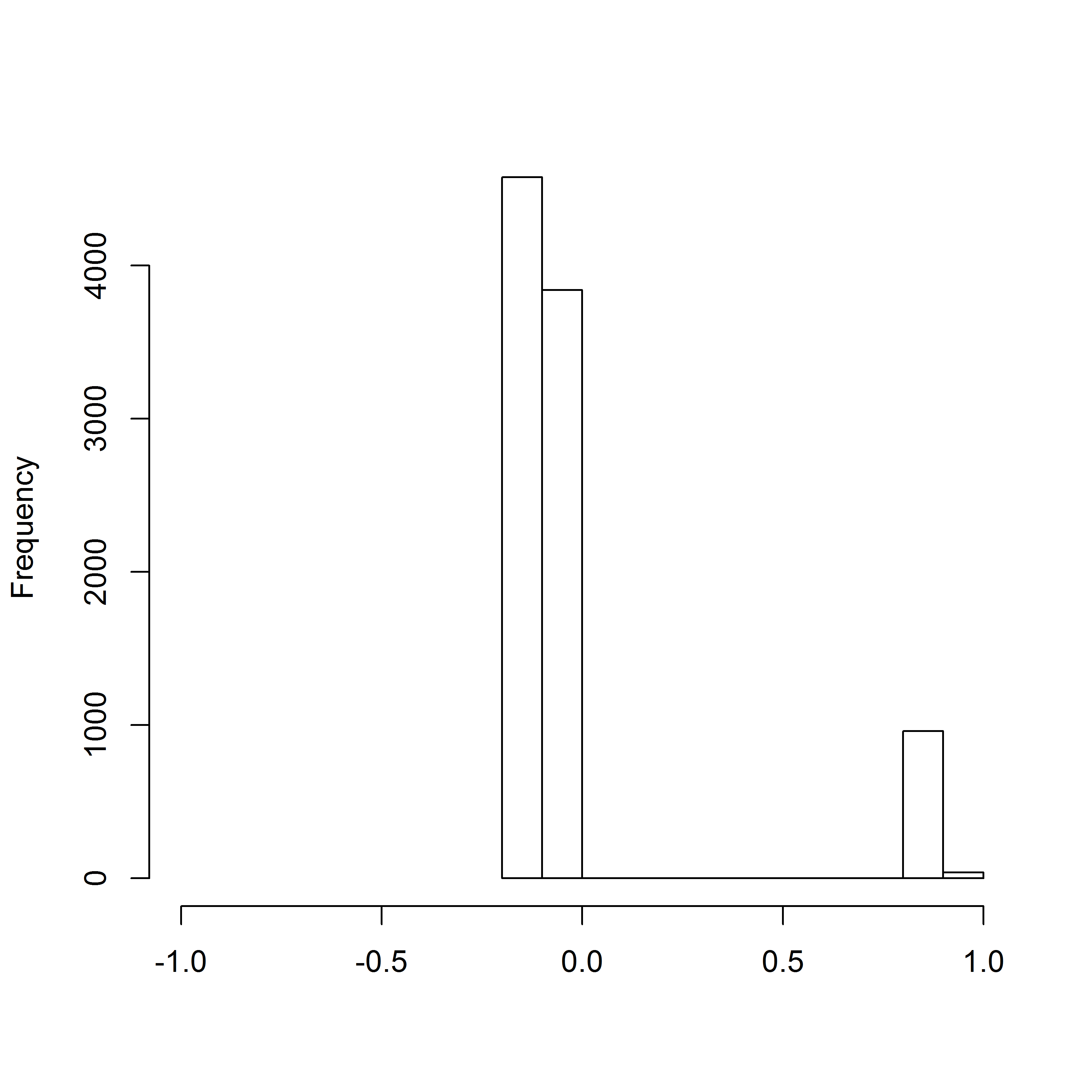

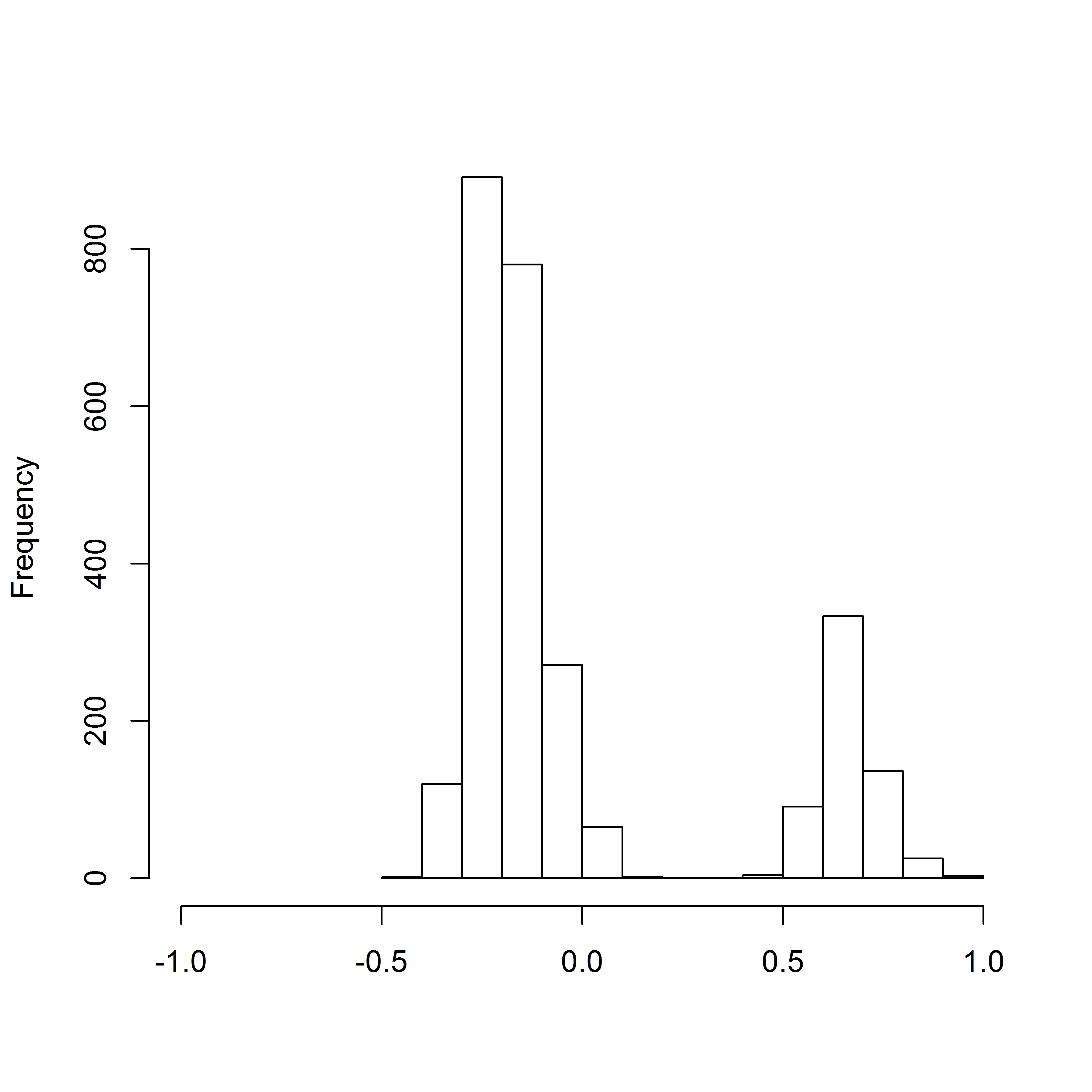

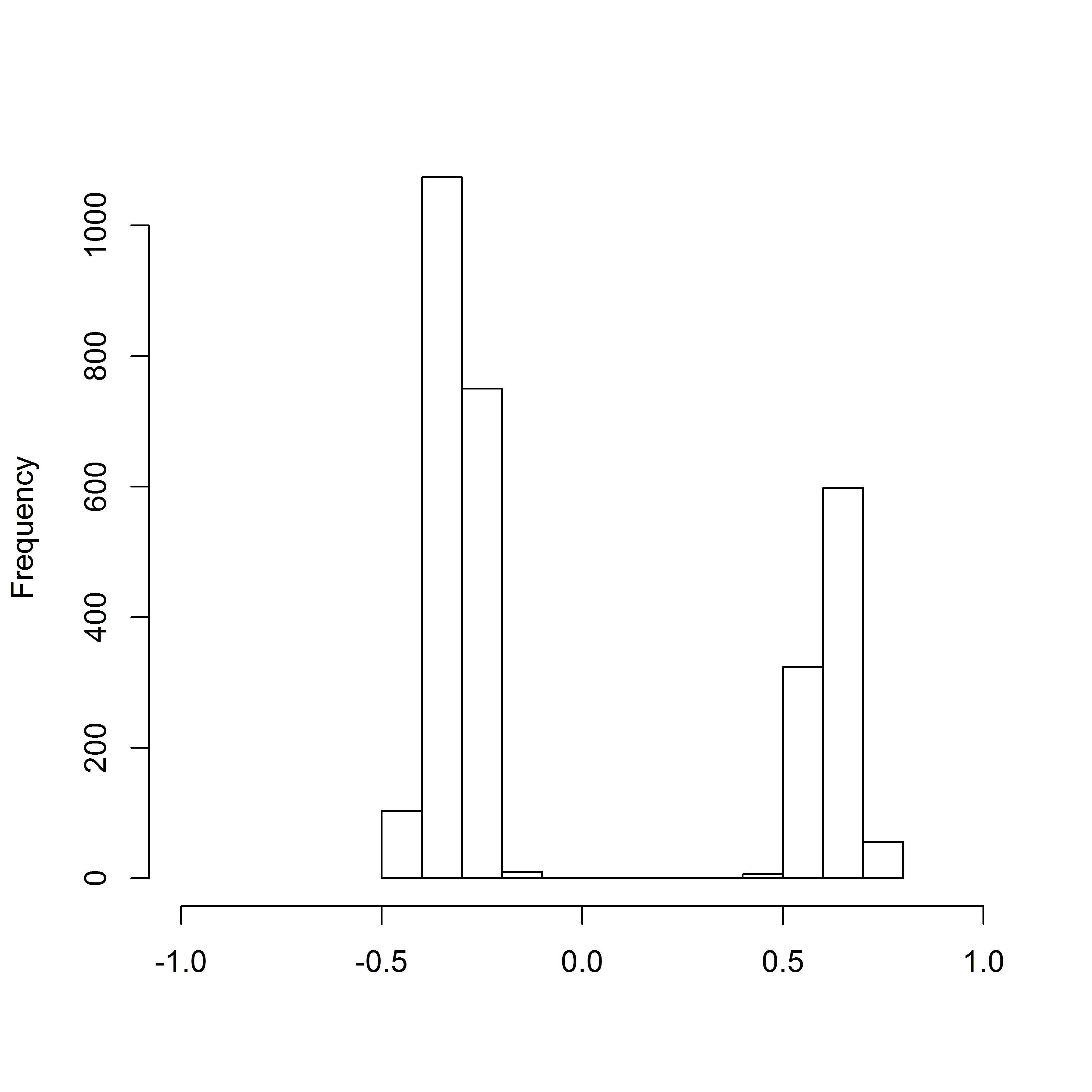


Supplementary Figure S3: Histograms of the residuals on the liability scale of the combined data (top left), the UK (top right), the US (bottom left) and the SEE (bottom right) cohorts.


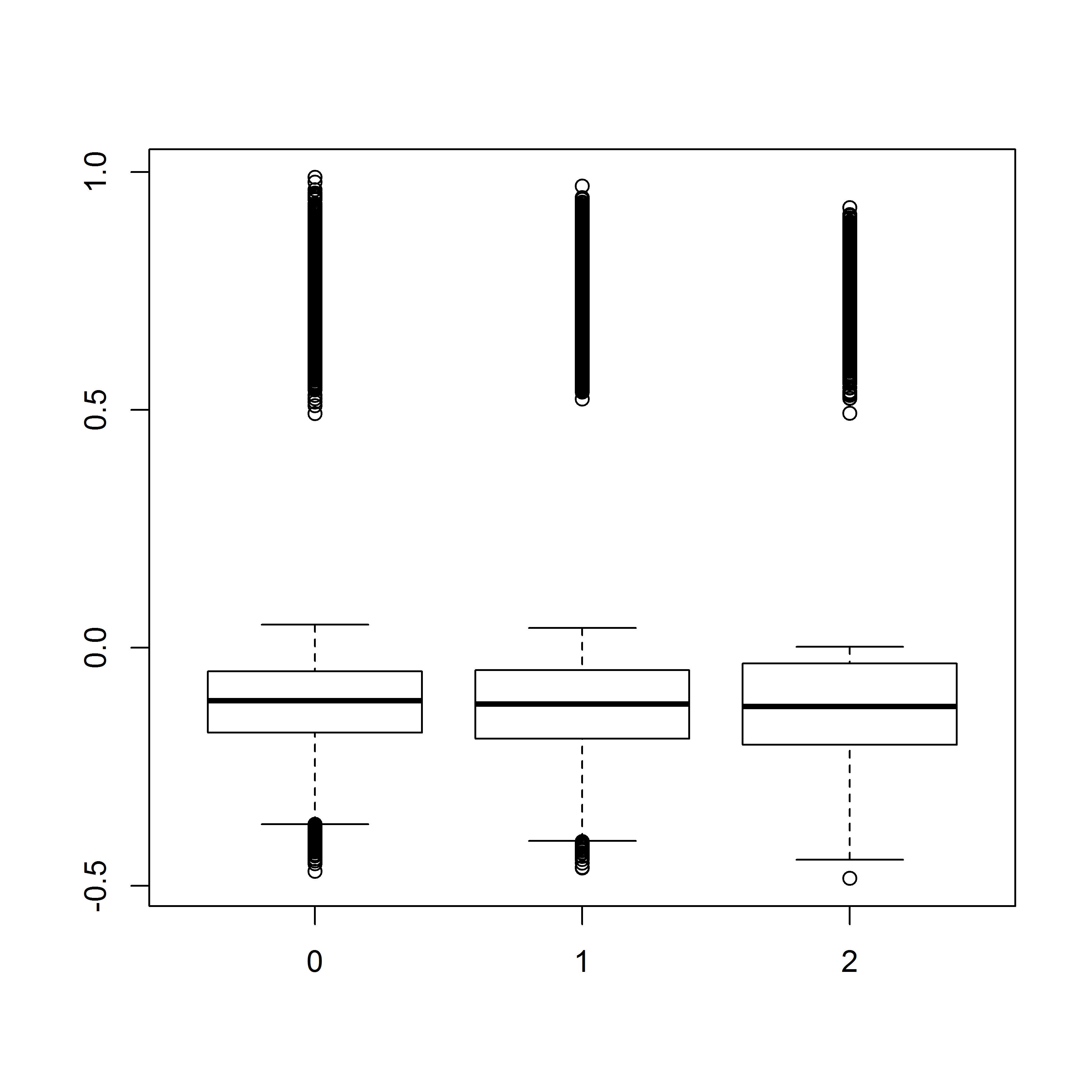

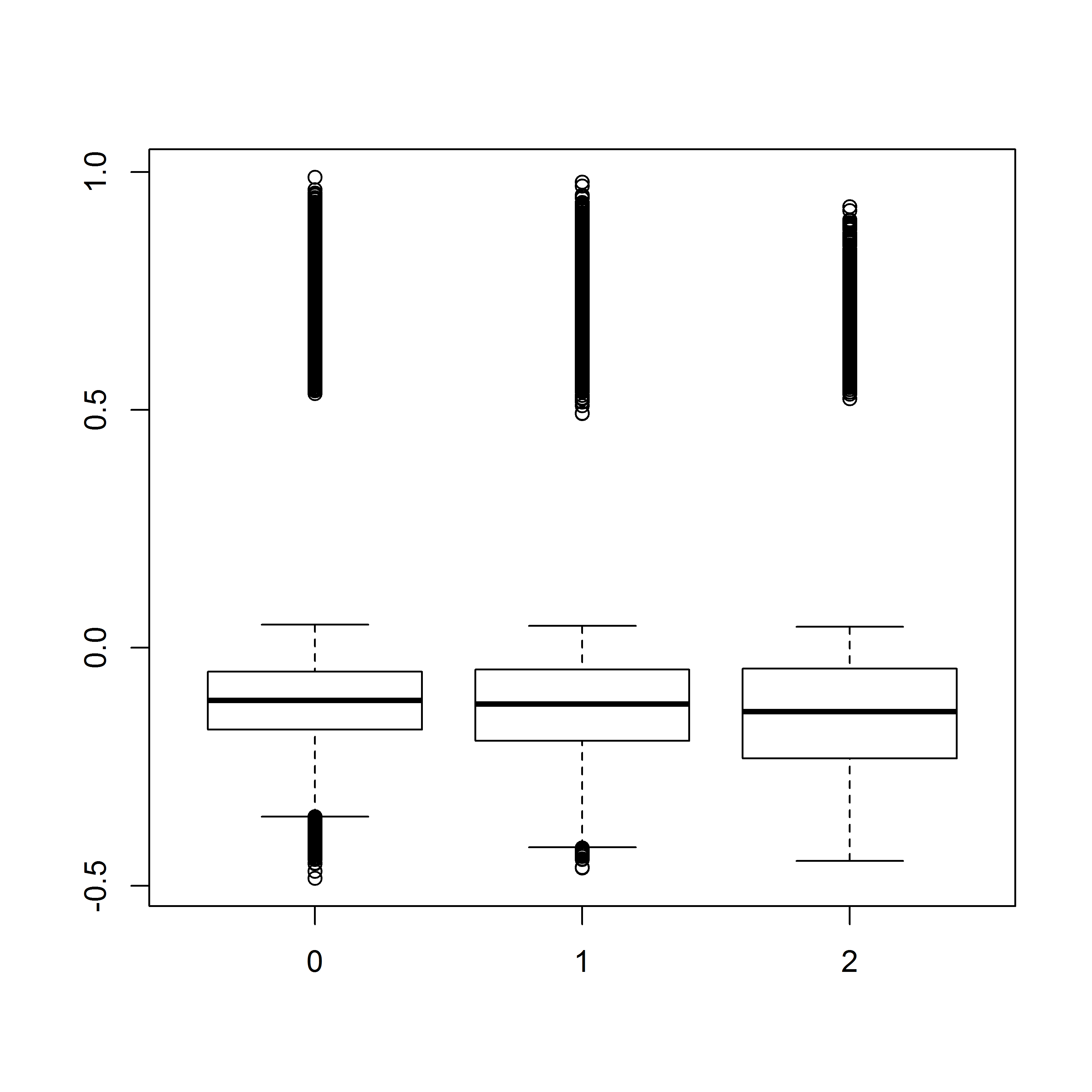

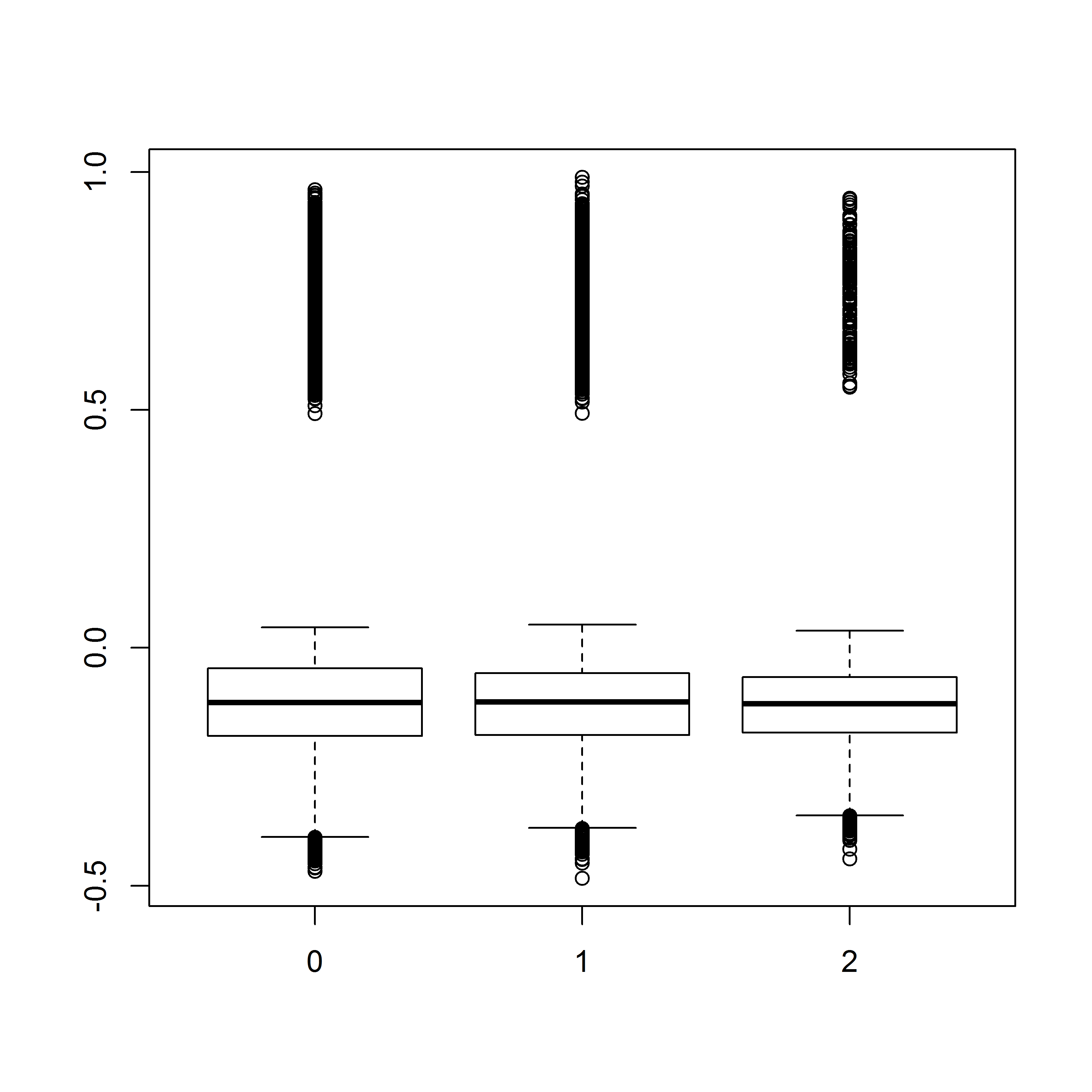

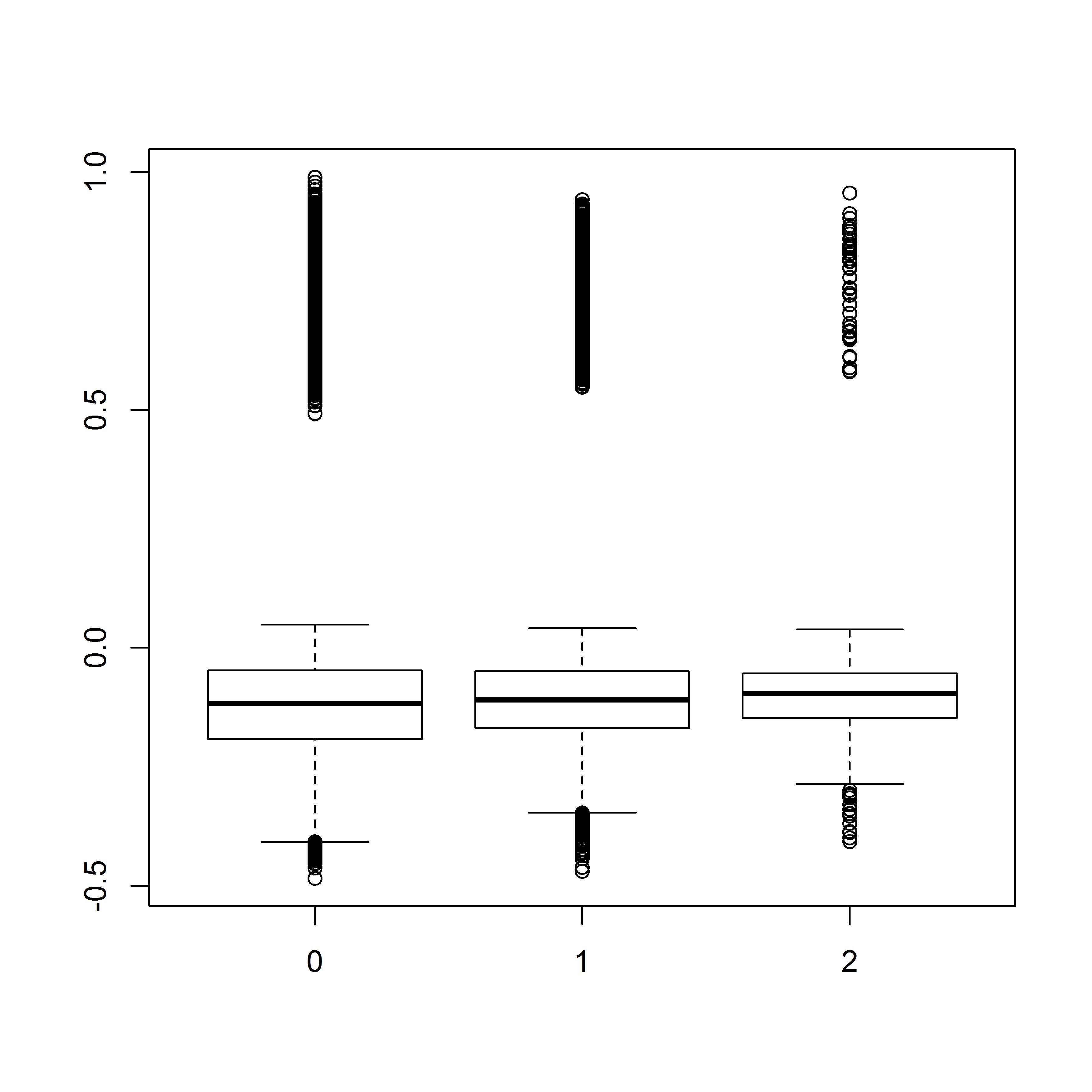


Supplementary Figure S4: Box plots of the residuals on the liability scale in the three genotypes of vGWAS signals rs9275428 (*HLA-DQB1*, top left), rs2852853 (*DHCR7*, top right), rs71624119 (*ANKRD55*, bottom left) and rs62389423 (*IRF4*, bottom right). The ‘0’, ‘1’ and ‘2’ on the x axis representing three genotypes per SNP where ‘1’ represents the heterozygous genotype.

Supplementary Table S1: Results of cross validation tests of the vGWAS loci identified in the combined data*

| iteration | rs2852853_*DHCR7* | | rs9275428_*HLA-DQB1* | | rs71624119_*ANKRD55* | | rs62389423_*IRF4* | |
| --- | --- | --- | --- | --- | --- | --- | --- | --- |
|  | set1 | set2 | set1 | set2 | set1 | set2 | set1 | set2 |
| 1 | 1.8E-04 | **4.2E-11** | **2.7E-16** | **9.9E-09** | **2.3E-11** | 2.1E-05 | 4.9E-04 | 6.5E-05 |
| 2 | **1.5E-08** | 6.4E-06 | **1.3E-10** | **4.1E-13** | 4.0E-06 | **3.8E-10** | 1.8E-04 | 1.5E-04 |
| 3 | 8.2E-08 | 5.9E-07 | **6.2E-13** | **4.9E-11** | 5.8E-06 | **4.8E-10** | 1.7E-04 | 2.9E-05 |
| 4 | **4.2E-09** | 1.1E-05 | **3.9E-17** | **3.3E-08** | 1.0E-07 | **2.8E-08** | 1.2E-03 | 5.9E-05 |
| 5 | 4.1E-06 | **3.4E-08** | **1.1E-10** | **9.8E-14** | 1.1E-07 | **2.1E-08** | 4.3E-05 | 4.1E-04 |
| 6 | **7.6E-10** | 2.8E-05 | **1.7E-10** | **6.9E-14** | 2.8E-06 | **2.3E-09** | 1.4E-07 | 3.3E-02 |
| 7 | 1.8E-04 | **1.0E-10** | **7.9E-11** | **2.8E-13** | **4.2E-08** | **3.9E-08** | 2.3E-02 | 2.6E-07 |
| 8 | 2.5E-06 | **1.8E-08** | **5.4E-14** | **6.0E-11** | **7.2E-09** | 2.4E-07 | 1.3E-04 | 3.5E-04 |
| 9 | 2.2E-06 | **2.6E-08** | **4.2E-08** | **2.8E-17** | 7.1E-06 | **3.7E-10** | 1.1E-03 | 2.5E-05 |
| 10 | **1.2E-08** | 2.3E-06 | **1.3E-18** | 9.5E-07 | 6.0E-08 | **3.1E-08** | 7.0E-05 | 6.0E-04 |

*: genome-wide significant in bold; Immunochip significant underlined
